# Novobiocin inhibits membrane synthesis and vacuole formation of *Enterococcus faecalis* protoplasts

**DOI:** 10.15698/mic2020.11.735

**Published:** 2020-08-10

**Authors:** Rintaro Tsuchikado, Satoshi Kami, Sawako Takahashi, Hiromi Nishida

**Affiliations:** 1 Department of Biotechnology, Toyama Prefectural University, Japan.

**Keywords:** bacterial protoplasts/spheroplasts, DNA replication, Enterococcus faecalis, novobiocin, plasma membrane synthesis, vacuole formation

## Abstract

We demonstrate that plasma membrane biosynthesis and vacuole formation require DNA replication in *Enterococcus faecalis* protoplasts. The replication inhibitor novobiocin inhibited not only DNA replication but also cell enlargement (plasma membrane biosynthesis) and vacuole formation during the enlargement of the *E. faecalis* protoplasts. After novobiocin treatment prior to vacuole formation, the cell size of *E. faecalis* protoplasts was limited to 6 μm in diameter and the cells lacked vacuoles. When novobiocin was added after vacuole formation, *E. faecalis* protoplasts grew with vacuole enlargement; after novobiocin removal, protoplasts were enlarged again. Although cell size distribution of the protoplasts was similar following the 24 h and 48 h novobiocin treatments, after 72 h of novobiocin treatment there was a greater number of smaller sized protoplasts, suggesting that extended novobiocin treatment may inhibit the re-enlargement of *E. faecalis* protoplasts after novobiocin removal. Our findings demonstrate that novobiocin can control the enlargement of *E. faecalis* protoplasts due to inhibition of DNA replication.

## INTRODUCTION

DNA replication is essential for cell division in normally dividing bacterial cells (native forms) [1] and affects both cell morphology and cell division [2–4]. It has been reported that the bacterial chromosome DNA attaches to the plasma membrane [5–10] although cell division does not occur in bacterial protoplasts or spheroplasts in the presence of an inhibitor of peptidoglycan biosynthesis [11–14]; removal of the peptidoglycan synthetic inhibitor can induce recovery from *Escherichia coli* spheroplasts to their native forms [15–17]. However, spheroplasts can enlarge in Difco Marine Broth (DMB) containing a peptidoglycan biosynthesis inhibitor, for example, penicillin [18–21], and interestingly, DNA replication has been reported during protoplast or spheroplast enlargement [13, 14, 18, 19, 23]. In the protoplasts of the gram-positive lactic acid bacterium *Enterococcus faecalis*, the amount of DNA increased during the protoplast enlargement between 0 and 96 h of incubation [23]. In addition, when the DNA replication inhibitor novobiocin was added to the medium after 24 h of incubation, the *E. faecalis* protoplast enlargement, as well as DNA replication was inhibited. Direct injection of DNA into spheroplasts that are larger than 15 µm in diameter also leads to vacuole formation [13, 14, 22, 23].

The bacterial cell division cycle may have several checkpoints [24–26]; although bacterial protoplasts do not divide, they can enlarge. Therefore, during protoplast enlargement, the bacterial cell division checkpoints of the native forms cannot function. In this study, we measured the increase in *E. faecalis* protoplast size and DNA amount, and also added novobiocin at different incubation times to test its effects. Without novobiocin treatment, *E. faecalis* protoplasts form vacuoles that also expand during the protoplast enlargement [23]. In this study, we focused on the relation between vacuole formation and the DNA replication.

## RESULTS

### Both DNA replication and cell enlargement of *E. faecalis* protoplasts stopped at 120 h of incubation

**Figure 1:**
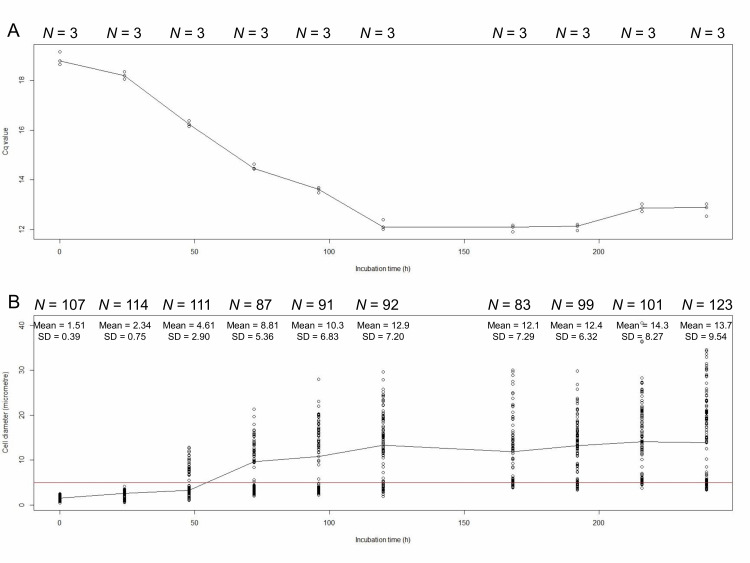
FIGURE 1: Cq values (A) and cell diameters (mm; B) of *E. faecalis* protoplasts at 0, 24, 48, 72, 96, 120, 168, 192, 216, and 240 h of incubation. We used *dnaA* for qPCR. The red line indicates that the diameter of cells is 5 μm.

We measured the amount of *E. faecalis* chromosomal DNA by using real-time quantitative PCR (qPCR). The quantification cycle (Cq) value decreases with increasing DNA concentration. qPCR showed that the Cq values decreased from 0 h to 120 h of incubation of *E. faecalis* protoplasts in DMB plus penicillin (**Fig. 1A**, **Table 1** upper part). Cell diameters increased with 0 h to 120 h of incubation (**Fig. 1B**, **Table 1** lower part). The Cq values and cell diameters at 120, 168, 192, 216, and 240 h of incubation did not significantly (*p* > 0.05) differ (**Tables 1** and **2**). The DNA replication and cell enlargement of *E. faecalis* protoplasts stopped at 120 h of incubation in DMB containing penicillin, with no further significant change in DNA amount and cell size.

**TABLE 1.** Pairwise comparisons of novobiocin treatment.

**Pairwise comparisons of Cq values using t test with non-pooled SD (p value adjustment by Bonferroni).**									
	**0 h**	**24 h**	**48 h**	**72 h**	**96 h**	**120 h**	**168 h**	**192 h**	**216 h**
**24 h**	1								
**48 h**	0.03843	0.00435							
**72 h**	0.00977	0.00042	0.00221						
**96 h**	0.00637	0.0002	0.0004	0.02576					
**120 h**	0.00033	0.00017	0.00189	0.01182	0.06034				
**168 h**	0.00148	4.10E-05	0.00013	0.00104	0.00646	1			
**192 h**	0.00241	5.30E-05	8.00E-05	0.00069	0.00446	1	1		
**216 h**	0.00152	7.50E-05	0.00048	0.0075	0.15061	0.4254	0.10069	0.11339	
**240 h**	0.00039	0.00162	0.01579	0.1158	0.9473	1	0.84134	1	1
**Pairwise comparisons of cell diameters using t test with non-pooled SD (p value adjustment by Bonferroni).**									
	**0 h**	**24 h**	**48 h**	**72 h**	**96 h**	**120 h**	**168 h**	**192 h**	**216 h**
**24 h**	< 2E-16								
**48 h**	< 2E-16	3.70E-11							
**72 h**	< 2E-16	< 2E-16	4.80E-08						
**96 h**	< 2E-16	< 2E-16	9.40E-10	1					
**120 h**	< 2E-16	< 2E-16	< 2E-16	0.0014	0.6284				
**168 h**	< 2E-16	< 2E-16	1.20E-12	0.0463	1	1			
**192 h**	< 2E-16	< 2E-16	< 2E-16	0.0021	1	1	1		
**216 h**	< 2E-16	< 2E-16	< 2E-16	6.30E-06	0.0129	1	1	1	
**240 h**	< 2E-16	< 2E-16	< 2E-16	0.0002	0.1207	1	1	1	1

### Novobiocin inhibits DNA replication without degradation

**Figure 2:**
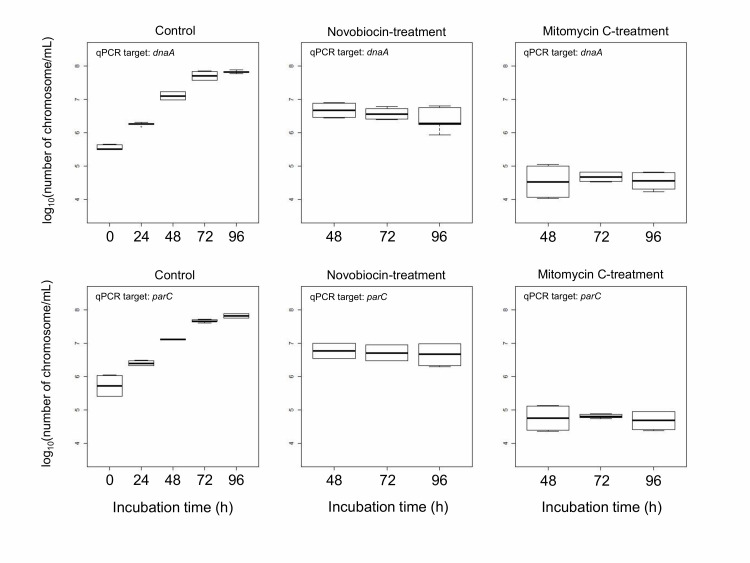
FIGURE 2: Boxplot of DNA concentration of *E. facalis* protoplasts. Control protoplasts were not treated by any DNA replication inhibitors. Mitomycin C or novobiocin was added at 24 h of incubation. After that, mitomycin C- and novobiocin-treated protoplasts were used at 48 (24 h-treatment), 72 (48 h-treatment), and 96 h (72 h-treatment) of incubation. Each experiment was performed with 8 replicates.

We compared DNA concentration of mitomycin C-treated protoplasts and novobiocin-treated protoplasts. The DNA amount was measured using real-time qPCR with two primer sets that amplified *dnaA* near the replication initiation site and *parC* near the termination site [23]. The result indicated that the DNA concentration of novobiocin-treated protoplasts was between those of control protoplasts at 24 h and 48 h of incubation (**Fig. 2**). On the other hand, the DNA concentration of mitomycin C-treated protoplasts was below that of control protoplasts at 0 h of incubation (**Fig. 2**). These results indicate that mitomycin C degraded the *E. faecalis* chromosomal DNA but novobiocin did not (**Fig. 2**). The DNA degradation by mitomycin C was reported [27, 28].

### Novobiocin treatment between 24 and 48 h of incubation increases the number of cells with a diameter of > 5 μm without vacuoles

**Figure 3:**
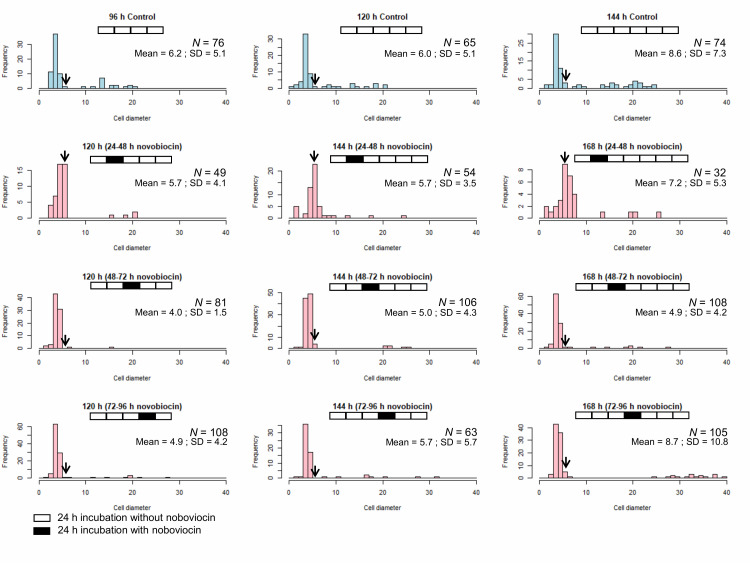
FIGURE 3: Size distribution of the control *E. faecalis* protoplast cells and *E. faecalis* protoplast cells treated with novobiocin for 24 h. Arrows indicate the fraction of cells with a diameter between 5 and 6 μm.

We showed that novobiocin treatment inhibited the *E. faecalis* protoplast enlargement [23]. Here, we investigated the effect of novobiocin treatment at different incubation times on the protoplast enlargement. Without novobiocin treatment (control), the cell diameters of *E. faecalis* protoplasts showed a peak between 3 and 4 μm at 96, 120, and 144 h of incubation (the first row in **Fig. 3**). The cell diameter distributions following novobiocin treatment for 48 and 72 h, and 72 and 96 h (the third and fourth rows in **Fig. 3**, respectively) were similar to that seen in case of the control. However, the cell diameter distribution following novobiocin treatment for 24 and 48 h (the second row in **Fig. 3**) peaked between 5 and 6 μm (shown with arrows in **Fig. 3**) at 120, 144, and 168 h of incubation.

Next, we compared cells of *E. faecalis* protoplasts at 120 h of incubation without novobiocin treatment (the first row and the second column in **Fig. 3**) and those of 144 h of incubation with novobiocin treatment (the second, third, and fourth rows and the second column in **Fig. 3**). The number of cells with vacuoles was 15 (23% of 65 cells), five (9.3% of 54 cells), six (5.7% of 106 cells), and eight (13% of 63 cells), in 120 h incubation-protoplasts without novobiocin treatment, 144 h incubation-protoplasts with 24 to 48 h novobiocin treatment, 144 h incubation-protoplasts with 48 to 72 h novobiocin treatment, and 144 h incubation-protoplasts with 72 to 96 h novobiocin treatment, respectively (**Table 2**). In addition, the number of cells with a diameter of > 5 μm without vacuoles was one (1.5% of 65 cells), 29 (54% of 54 cells), four (3.8% of 106 cells), and zero (0% of 63 cells), in 120 h incubation-protoplasts without novobiocin treatment, 144 h incubation-protoplasts with 24 to 48 h novobiocin treatment, 144 h incubation-protoplasts with 48 to 72 h novobiocin treatment, and 144 h incubation-protoplasts with 72 to 96 h novobiocin treatment, respectively (**Table 2**).

**TABLE 2.** Number of cells with vacuoles and cells with a diameter of > 5 μm without vacuoles.

**Cell type**	**120 h incubation without novobiocin**	**144 h incubation with 24-48 h novobiocin**	**144 h incubation with 48-72 h novobiocin**	**144 h incubation with 72-96 h novobiocin**
Cells with vacuoles	15 (23.1%)	5 (9.3%)	6 (5.7%)	8 (12.7%)
Cells with a diameter of > 5 μm without vacuoles	1 (1.5%)	29 (53.7%)	4 (3.8%)	0 (0.0%)
Others	49 (75.4%)	20 (37.0%)	96 (90.6%)	55 (87.3%)
Total	65 (100%)	54 (100%)	106 (100%)	63 (100%)

### Novobiocin treatment duration influences protoplast enlargement in the presence of novobiocin and re-enlargement after novobiocin removal

**Figure 4:**
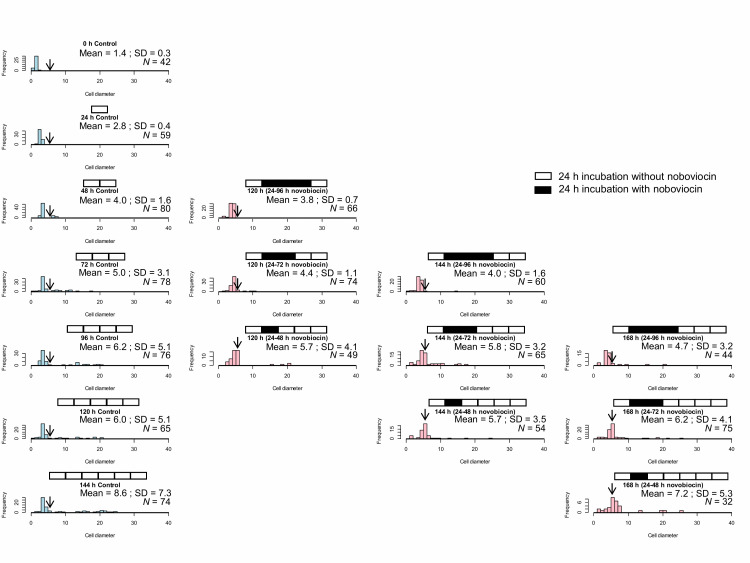
FIGURE 4: Size distribution of control *E. faecalis* protoplast cells and *E. faecalis* protoplast cells treated with novobiocin for different time periods (24, 48, and 72 h). The arrows indicate the fraction of cells with a diameter between 5 and 6 μm.

Here, the effect of novobiocin treatment time on *E. faecalis* protoplasts after novobiocin removal was investigated. Comparison among cell size distributions at the same incubation times including the time of novobiocin treatment, for example, 120 h (24–96 h novobiocin), 120 h (24–72 h novobiocin), and 120 h (24–48 h novobiocin), as shown in in **Fig. 4**, demonstrates that an extended novobiocin treatment time leads to a smaller cell size. Kolmogorov-Smirnov test showed that the cell size distribution at 120 h (24–72 h novobiocin) was significantly different (*p* < 0.05) from that at 120 h (24–96 h novobiocin) and that at 120 h (24–48 h novobiocin). Therefore, cell enlargement was inhibited by the novobiocin treatment.

Comparison between cell size distributions at the same incubation time with different novobiocin treatments, for example, 120 h (24–48 h novobiocin), 144 h (24–72 h novobiocin), and 168 h (24–96 h novobiocin), as shown in **Fig. 4**, also indicates that the longer the novobiocin treatment duration, the smaller the cell size. However, the cell size distribution at 144 h (24–72 h novobiocin) was significantly different (*p* < 0.05) from that at 168 h (24–96 h novobiocin), but not (*p* > 0.05) from that at 120 h (24–48 h novobiocin). In addition, the cell size distribution at 144 h (24–48 h novobiocin) was not significantly different from that at 168 h (24–72 h novobiocin). These results indicate that the cell size distribution was not significantly different between the cells treated with novobiocin for 24 h and those treated with novobiocin for 48 h.

**Figure 5:**
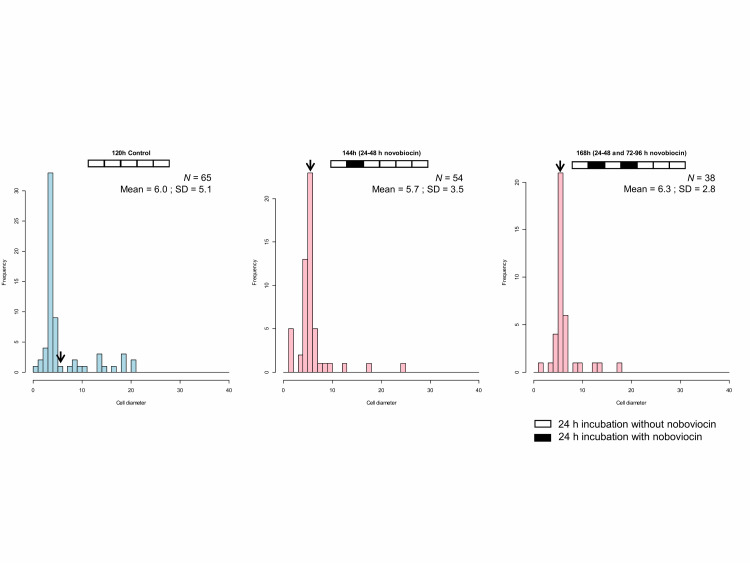
FIGURE 5: Cell size distribution of *E. faecalis* protoplast cells treated twice with novobiocin. The arrows indicate the fraction of cells with a diameter between 5 and 6 μm.

When *E. faecalis* protoplasts were incubated for 168 h with two novobiocin treatments (24–48 h and 72–96 h), the cell size distribution (**Fig. 5**) was similar to that of 144 h (24–48 h novobiocin) of **Fig. 4**. Kolmogorov-Smirnov testing showed that the cell size distribution of 168 h (24–48 h and 72–96 h novobiocin) was not significantly different (*p* > 0.05) from that of 144 h (24–48 h novobiocin). Thus, the cell size distribution of 168 h incubation with two separated novobiocin treatments has a peak between 5 and 6 μm (**Fig. 5**), indicating that the time of novobiocin treatment does not influence the cell enlargement.

## DISCUSSION

In our previous study, we measured the amount of *E. faecalis* DNA and cell size between 0 and 96 h of incubation with novobiocin [23]. However, it was uncertain when the enlargement of the *E. faecalis* protoplasts stopped. This study demonstrates that DNA replication and cell enlargement of *E. faecalis* protoplasts stopped at the same time, at 120 h of incubation in DMB containing penicillin (**Fig. 1**), and suggests that plasma membrane biosynthesis requires DNA replication. We have shown that lipid composition changed during enlargement of *Deinococcus grandis* spheroplasts [21], indicating that membrane biosynthesis occurs in enlargement. In the native forms of bacteria, DNA replication should occur before the cell division; bacterial protoplasts, however, cannot divide in the presence of penicillin. In the presence of penicillin, *Escherichia coli* forms bulges at sites where the new cell wall is normally formed [2]. This bulge formation also requires DNA replication [2] and is consistent with our findings. It is likely that there is a mechanism that connects DNA replication and plasma membrane biosynthesis, which we do not yet know. Although more work is needed to elucidate how DNA replication and plasma membrane biosynthesis are connected, the bacterial protoplast enlargement system is a highly useful tool for the studies.

In this study, we used novobiocin as an inhibitor of DNA replication. Novobiocin inhibits DNA supercoiling catalyzed by DNA gyrase [29, 30]. Following novobiocin addition to *E. faecalis* protoplasts at 24 h incubation in DMB containing penicillin, the cell enlargement is strongly inhibited, but the cells are alive, lack vacuoles, and grow very slowly [23]. The cell size distribution of the *E. faecalis* protoplasts with novobiocin treatment between 24 and 48 h of incubation was unique with a peak of 5–6 μm (**Fig. 3**) at 96, 120, and 144 h of incubation.

Usually, during *E. faecalis* protoplast enlargement, cells with a diameter of > 5 μm are observed after 48 h of incubation (**Fig. 1B**), all of which have vacuoles [23]. However, the number of cells with vacuoles is the lowest in case of the *E. faecalis* protoplasts that were treated with novobiocin for 24–48 h. This suggests that vacuole formation cannot occur after removal of novobiocin from protoplasts treated with novobiocin for 24–48 h, but some plasma membrane biosynthesis can still occur and the cells with a diameter of > 5 μm without vacuoles are observed. If protoplasts form vacuoles before 24 h, they may continue to form vacuoles after novobiocin removal. This is consistent with the fact that a limited number of enlarged protoplasts with vacuoles were found in *E. faecalis* protoplasts with novobiocin treatment between 48 and 72 h, and between 72 and 96 h (**Table 2**). These results suggest that vacuole formation requires DNA replication as well as plasma membrane biosynthesis, which may be related to the fact that plasma membrane endocytosis occurred at the early stage of the vacuole formation [13]. We have recently reported that bacterial plasma and vacuolar membranes were synchronously biosynthesized with differences depending on the bacterial species [31], strongly suggesting that DNA replication may be associated with vacuole formation as well as plasma membrane synthesis.

*E. faecalis* protoplasts enlarge very slowly in the presence of novobiocin [23]. Comparison of cell size distributions among the protoplasts with the same incubation times before different novobiocin treatment times showed that the longer the novobiocin treatment duration, the smaller the cell size (**Fig. 4**). This indicates that the rate of enlargement following novobiocin removal is faster than that in the presence of novobiocin. In addition, comparison of cell size distributions among the protoplasts with the same incubation times after different novobiocin treatment times also showed that the longer the novobiocin treatment, the smaller the cell size (**Fig. 4**). This suggests that the increase in the duration of novobiocin treatment may inhibit the re-enlargement of the *E. faecalis* protoplasts after novobiocin removal or results in a greater amount of time being required to reset the cell system.

DNA replication-dependent vacuole formation may play the role of a checkpoint for the *E. faecalis* protoplast enlargement. Although untreated protoplasts without vacuoles are up to 5 μm in diameter, the cell size of the novobiocin treated protoplasts without vacuoles is up to 6 μm in diameter demonstrating unchecked enlargement as novobiocin inhibits DNA replication-dependent plasma membrane biosynthesis. In our laboratory, we have microinjected DNA or proteins to enlarged bacterial protoplasts, but we have not yet controlled these protoplast conditions [31]. In the near future, we will be able to control the protoplast cell size using novobiocin.

## MATERIALS AND METHODS

### Preparation and culture of protoplasts

*E. faecalis* NBRC 100480 was cultivated and protoplasts were prepared as previously described [23]. The protoplasts were centrifuged at 7000 rpm for 5 min and resuspended in DMB (5 g/L peptone, 1 g/L yeast extract, 0.1 g/L ferric citrate, 19.45 g/L NaCl, 5.9 g/L MgCl_2_, 3.24 g/L MgSO_4_, 1.8 g/L CaCl_2_, 0.55 g/L KCl, 0.16 g/L NaHCO_3_, 0.08 g/L KBr, 34 mg/L SrCl_2_, 22 mg/L H_3_BO_3_, 8 mg/L Na_2_HPO_4_, 4 mg/L Na_2_SiO_3_, 2.4 mg/L NaF, and 1.6 mg/L NH_4_NO_3_ [BD, Franklin Lakes, NJ]) containing 300 µg/mL penicillin G. The resulting suspension (5 µL) was diluted with 1 mL of DMB containing 300 µg/mL penicillin G (Wako, Osaka) and incubated at 24 °C.

### Quantitative PCR (qPCR)

DNA was extracted from the culture (1 mL) and purified using a NucleoSpin Tissue XS kit (Macherey-Nagel GmbH & Co. KG, Düren). From the resulting 15 µL of each DNA solution, 1 µL was used for qPCR amplification for *dnaA* and *parC* [23]. DNA was amplified with FastStart Essential DNA Green Master kit (Roche, Basel) on a LightCycler Nano system (Roche). qPCR was performed at 95°C for 600 s followed by 45 cycles of denaturation (95°C for 10 s), annealing (55°C for 10 s), and extension (72°C for 15 s). After extension, a melting curve analysis was performed from 60°C–95°C at 0.1°C/s to confirm that nonspecific products had not been generated. The Cq values were obtained using LightCycler Nano software (Roche). The Cq values were estimated by pairwise *t* test using the statistical software R.

### Cell size measurement

Phase-contrast microscopy images of the protoplasts were obtained using an Olympus CKX41 (Tokyo). The cell sizes were measured using cellSens Standard 1.11 imaging software (Olympus, Tokyo). The cell sizes were estimated by pairwise *t* test and Kolmogorov-Smirnov test using the statistical software R (https://www.r-project.org/).

### Novobiocin treatment

Novobiocin (Wako, Osaka) was added to the incubation medium at 24, 48, and 72 h at a final concentration of 50 µg/mL. It was not added to the control. We removed novobiocin from the incubation medium at 48, 72, and 96 h, respectively. Then, we determined the cell enlargement and measured the cell size at 120, 144, and 168 h of incubation. Cultures were incubated in novobiocin for 48 and 72 h by adding novobiocin at the 24 h timepoint and then removing novobiocin at 72 and 96 h, respectively.

When an addition of novobiocin was performed twice, novobiocin was first added at a final concentration of 50 µg/mL at the 24 h time point; novobiocin was then removed from the incubation medium at 48 h. Novobiocin was added a second time at 72 h and finally removed at 96 h. Cell enlargement was determined by measuring the cell size at 168 h of incubation. Novobiocin removal was performed by 50-fold dilution using filtered DMB containing penicillin.

### Mitomycin C treatment

Mitomycin C (Sigma) was added to the incubation medium at 24 h at a final concentration of 25 µg/mL. It was not added to the control.

## Abbreviations:

Cq: – quantification cycle,
qPCR: – quantitative PCR.
